# Association between functional variant of inflammatory system gene (PSMA6) and end-stage kidney disease

**DOI:** 10.1007/s11255-016-1420-y

**Published:** 2016-09-26

**Authors:** Monika Buraczynska, Anna Stec, Aleksandra Filipczak, Andrzej Ksiazek

**Affiliations:** Department of Nephrology, Medical University of Lublin, Jaczewskiego 8, 20-954 Lublin, Poland

**Keywords:** End-stage renal disease, Genotyping, LVH, PSMA6, Single nucleotide polymorphism

## Abstract

**Background:**

The proteasome system is involved in several disorders. The 5′ untranslated region of PSMA6 gene contains a single nucleotide polymorphism (SNP) −8 C/G, associated with diabetes, myocardial infarction and coronary artery disease.

**Methods:**

We examined 584 patients with end-stage kidney disease (ESKD) and 430 controls. All were genotyped for −8 C/G SNP by polymerase chain reaction and restriction analysis.

**Results:**

We observed lower frequency of CG + GG genotypes in patients than in controls (20 vs. 42 %, *p* = 0.0038). The odds ratio of 0.34 (95 % CI 0.26–0.45) suggests association of CG + GG with decreased risk of ESKD. We investigated the association between PSMA6 polymorphism and LVH present in 54 % of patients. There was a significant association of CG + GG genotype with LVH, with over 75 % of CG + GG in patients with LVH. This effect was independent from other common causes of LVH—age (OR 1.12, *p* = 0.643) and hypertension (OR 1.72, *p* = 0.422).

**Conclusion:**

We demonstrated for the first time that PSMA6 polymorphism might be a protective factor for ESKD. On the other hand, CG + GG genotypes are independently related to LVH in ESKD patients.

## Introduction

End-stage kidney disease (ESKD) is a complex phenotype resulting from underlying kidney disease and interacting genetic and environmental factors. Despite rapid improvements in dialysis technology, the mortality rate in dialyzed ESKD patients is very high [1, 2]. The identification of causative genes predisposing to chronic kidney disease and its complications could provide means to better understanding the pathogenesis of the disease and result in better prevention, diagnosis and treatment [3, 4].

The proteasome is a large multiple subunit enzyme complex that plays a central role in the degradation of intracellular proteins which control transcription rate, cell cycle progression and apoptosis [5, 6]. It regulates inflammatory processes and plays an important role in pathogenesis of human diseases such as cardiovascular diseases, diabetes, neurological diseases and cancer [7–10].

The PSMA6 gene is a single copy gene located on chromosome 14q13.2 [11]. It codes for a 246 residue protein called α1 that is structurally important in forming the outer α rings of the 20S core proteasome. The α1 protein function is also likely to be modulated by posttranslational modifications including phosphorylation, glycosylation and lysine acetylation [12]. The 5′ untranslated region of PSMA6 gene contains a single nucleotide polymorphism (SNP) −8 C/G (rs1048990) in exon 1. The risk conferring G allele enhances the transcription of PSMA6 [13]. Enhanced PSMA6 activity might exaggerate inflammation through activation of nuclear factor ĸB, a central transcription factor regulating expression of the genes of cytokines and adhesion molecules [14]. The −8 G/C polymorphism was reported to be associated with type 2 diabetes, myocardial infarction and coronary artery disease [13, 15–19].

In this preliminary case–control study, we investigated an association between functional polymorphism −8 C/G in PSMA6 gene and end-stage kidney disease.

## Patients and methods

### Subjects

The study population consisted of 584 unrelated, consecutive adult patients on maintenance dialysis. All patients were Caucasians of Polish origin. Chronic kidney disease was diagnosed according to KDOQI (Kidney Disease Outcomes Quality Initiative) definition. ESKD was defined as eGFR < 15 ml/min/1.73 m^2^ associated with clinical signs of uremic syndrome requiring dialysis. ESKD resulted mainly from primary chronic glomerulonephritis (*n* = 183), diabetic nephropathy (*n* = 132), interstitial nephritis (*n* = 71) and polycystic kidney disease (*n* = 45).

Cardiovascular disease was documented in 384 patients, as one or the combination of several pathological states: congestive heart failure, left ventricular hypertrophy, angina pectoris, ischemic heart disease, myocardial infarction, cerebral stroke or atheromatous lesions. There was a substantial overlap between categories. Left ventricular hypertrophy (LVH) was diagnosed by electrocardiography (ECG) and echocardiography. At the beginning of study, 426 patients were hypertensive and receiving antihypertensive medications. Hypertension was defined as a systolic blood pressure >140 mm Hg and diastolic blood pressure >90 mm Hg and/or use of antihypertensive medication.

Healthy control subjects (*n* = 430) were recruited mostly among hospital staff and blood bank donors who underwent health examination. All had normal electrocardiogram (ECG) and no clinical signs of CVD or renal disease. Subjects with a positive family history of renal or cardiovascular disease in first-degree relatives were excluded from the study.

An informed consent for participating in genetic studies was obtained from all ESKD and control subjects. The protocol of the study was approved by an institutional ethics committee. The investigation conforms to the principles of the Declaration of Helsinki.

### Determination of PSMA6 genotype

Genomic DNA was extracted from peripheral blood leukocytes prepared by the standard procedure and stored at −70 °C before use. The −8 C/G (rs1048990) polymorphism in the PSMA6 gene was analyzed by amplification of 343 bp DNA fragment by polymerase chain reaction (PCR). Genomic DNA (300 ng) was amplified using the following conditions: initial denaturation at 95 °C for 6 min, followed by 40 cycles of 94 °C for 30 s, annealing at 60 °C for 30 s and extension at 72 °C for 1 min. A final extension step was at 72 °C for 7 min. 10 μl of the PCR product was digested overnight at 37 °C with 5 U of Rsa I restriction endonuclease, and resulting fragments were separated on a 2.5 % agarose gel. The quality of genotyping was controlled by using blind DNA duplicates for random samples. In addition, 20 samples were randomly selected for each genotype and the PCR products were sequenced in CEQ8000 Genetic Analysis System (Beckman Coulter, England). There was a 100 % concordance between genotyping assays.

### Statistical analysis

Statistical calculations were performed using SPSS version 11.0 for Windows (SPSS, Inc., Chicago, IL, USA). For baseline characteristics, the normally distributed continuous variables are presented as mean ± SD. ANOVA, Pearson *Χ* ^2^ test and Mann–Whitney test were used for comparing discrete and continuous variables. The Hardy–Weinberg equilibrium was assessed using a *Χ* ^2^ test with 1 degree of freedom. Genotype distribution and allele frequencies were compared between groups using a Pearson *Χ* ^2^ test of independence with 2 × 2 contingency and *z* statistics. For significant allelic and genotyping associations, the adjusted odds ratios (OR) with corresponding 95 % confidence intervals (CI) were calculated. Power calculations were performed with the program of Purcell et al. (available at http://pngu.mgh.harvard.edu/~purcell/gpc/). In the ESKD patient group, the frequency of the PSMA6 G allele was 0.10. The study had 99.4 % power (*α* = 0.05) to detect an association (OR vs. controls 0.34, 95 % CI 0.26–0.45). Logistic regression analysis was performed to analyze the correlation of genotype and clinical characteristics, with adjustments for gender and age. Statistical significance was set at *p* < 0.05.

## Results

The genotypes of the −8 C/G PSMA6 gene polymorphism were determined in 584 ESKD patients and 430 healthy individuals. The demographic profile and clinical characteristics of studied ESKD patients and control subjects are summarized in Table 1. The ESKD patients presented an average age 6 years older than control subjects. They also had higher BMI as well as total cholesterol and triglyceride levels than controls.

**Table 1:** Demographic and clinical profile of studied subjects

Variables	ESRD patients	Controls	*p* value^a^
*N*	584	430	
Gender (M/F)	331/253	226/204	
Age at study (years)	56.2 ± 19	50 ± 17	<0.001
Years on dialysis	4.9 ± 3.3	NA	
Diabetes mellitus (%)	132 (23)	0	
Hypertension (%)	426 (73)	0	
BMI (kg/m^2^)	27.6 ± 4.5	26.3 ± 3.5	<0.001
Serum creatinine (μmol/l)	789.2 ± 112	ND	
Total cholesterol (mmol/l)	5.3 ± 1.81	3.8 ± 1.68	<0.001
HDL cholesterol (mmol/l)	1.2 ± 0.84	ND	
Triglycerides (mmol/l)	2.4 ± 1.73	1.16 ± 0.92^b^	<0.001

Values are presented as mean ± SD or numbers (%)

*ESRD* end-stage renal disease

^a^Where significant

^b^Triglycerides were determined in 23 % of control subjects

The frequencies of the genotypes and alleles in the control group were similar to those reported in other studies of European populations [16, 20, 21]. Table 2 presents the genotypes of the −8 C/G PSMA6 polymorphism in patients and controls. Since the numbers of individuals with the GG genotype were small, the carriers of the G allele (CG + GG) were combined into one group to increase the statistical power. The frequency of the CG + GG genotype was lower in ESKD patients than in controls (20 vs. 42 %, *p* = 0.0038). The odds ratio 0.34 (95 % CI 0.26–0.45) might suggest an association of the CG + GG genotype with decreased risk of end-stage kidney disease.

**Table 2:** Genotype and allele distribution of PSMA6 SNP in end-stage renal disease patients and controls

Subjects	−8 C/G genotype		MAF	*p* value	Adjusted OR (95 % CI)^b^ for CG + GG genotypes	*p* value
	CC	CG + GG^a^				
ESRD (*n* = 584)	467 (80)	117 (20)	0.10	0.0038	0.34 (0.26–0.45)	<0.0001
Controls (*n* = 430)	249 (58)	181 (42)	0.22		1.0 (reference)	

Genotype distributions are shown as numbers (%)

*ESRD* end-stage renal disease, *MAF* minor allele frequency

^a^The GG homozygotes were not analyzed separately due to their small number

^b^OR was adjusted for age, sex and body mass index. The nominal *p* values were adjusted according to Bonferroni correction

Left ventricular hypertrophy (LVH) is a strong cardiovascular risk marker in end-stage renal disease patients, so we also investigated the association between PSMA6 polymorphism and LVH present in 54 % of our patients (Table 3). We observed a strongly significant association of the CG + GG genotype with LVH, with over 75 % of all CG + GG genotypes found in patients with LVH. The logistic regression analysis showed that the effect of CG + GG genotype was independent from other common causes of LVH—age (OR 1.12, *p* = 0.643) and hypertension (OR 1.72, *p* = 0.422).

**Table 3:** Genotype and allele distribution of PSMA6 SNP in ESRD patients with and without LVH

Subjects	−8 C/G genotype		MAF	*p* value	OR (95 % CI)^b^ for CG + GG genotypes	*p* value
	CC	CG + GG^a^				
LVH (*n* = 315)	227 (72)	88 (28)	0.14	0.0002	3.21 (2.03–5.06)	<0.0001
Without LVH (*n* = 269)	240 (89)	29 (11)	0.05		1.0 (reference)	

Genotype distributions are shown as numbers (%)

*LVH* left ventricular hypertrophy, *MAF* minor allele frequency

^a^The GG homozygotes were not analyzed separately due to their small number

^b^OR was adjusted for age, sex and body mass index. The nominal *p* values were adjusted according to Bonferroni correction

## Discussion

The ubiquitin–proteasome system and its gene polymorphisms are extensively studied, mostly in the cardiovascular field [22]. The functional −8 G/C polymorphism in PSMA6 gene was earlier reported to be associated with several human diseases—type 2 diabetes, myocardial infarction and coronary artery disease [9, 16, 18, 23]. Our study was designed to address the potential involvement of the PSMA6 gene polymorphism for the first time in chronic kidney failure. In our group of patients with ESKD, the −8 G/C polymorphism was significantly associated with decreased risk of development of end-stage kidney disease. The PSMA6 genotype with a G allele might be a protective factor for the development of end-stage kidney disease. The mechanism of this protective effect needs to be elucidated, with considering also the influence of environmental and other coexisting genetic factors. There are two previous studies, reporting a decreased risk of stroke associated with the PSMA6 −8 G/C polymorphism in Caucasian populations [20, 21]. In both studies, the authors found a protective effect of the PSMA6 rs1048990 SNP on overall ischemic stroke, with OR 0.79, *p* = 0.037 and OR 0.80, *p* = 0.036, respectively. In the Chinese population, the PSMA6 rs1048990 CG + GG genotype and G allele were protective factors for coronary heart disease [17].

Left ventricular hypertrophy is one of the major cardiovascular complications of end-stage kidney failure. Left ventricular mass increases progressively with duration of dialysis treatment, even in normotensive patients [24]. Our study shows that the −8 C/G PSMA6 gene polymorphism is independently related to LVH in a population of ESKD patients. We observed that the presence of CG + GG genotypes conferred higher risk of the LVH (over threefold) than the presence of the CC genotype. There are other studies reporting gene variants related to LVH in patients on dialysis [25, 26]. Fedor et al. [27] reported an association of the D allele of the angiotensin-converting enzyme polymorphism with LVH development after renal transplantation. The end-stage kidney disease patients are subjected to pressure overload and several hemodynamic and non-hemodynamic factors. Thus, in this population of patients, it might be difficult to explain the mechanism of PSMA6 effect on LVH. Also, LVH is caused by lasting effects of several different mechanisms on myocardial anatomy and function [28]. The functional studies are needed to explore the mechanism of PSMA6 effect on LVH. Since the logistic regression analysis we performed showed that the effect of rs1048990 SNP on LVH was independent from hypertension, the possibility that it is secondary effect, through hypertension, is low.

As most of the association studies, ours has some potential limitations. A survival and selection bias cannot be excluded in this retrospective case–control study. To limit this bias, we included consecutive patients and tried to adjust for known confounding risk factors, but the comorbidities might still represent a confounding factor. The strengths of the study are the fairly large size of studied groups and that all patients and controls are of the same ethnic origin. There was also an adequate statistical power of the study for the type I error at 0.05 (99.4 % for ESKD vs. controls and 98.1 % for LVH vs. no LVH subgroups). All subjects were examined in a standardized manner, with well-defined diagnostic criteria, and genotyping was performed blind with respect to case–control status. However, the occurrence of kidney diseases, diabetes and comorbidities such as CVD depends on the interaction among the different risk alleles, environmental factors and the lifestyle. The influence of single polymorphisms is rather small, and an interactive effect of several factors may lead to an underestimation or overestimation of a role of given polymorphism in determining the phenotype. These results might not therefore apply to populations with different genetic or environmental background.

In conclusion, in this study we demonstrated for the first time that the PSMA6 gene polymorphism might be a protective factor for end-stage kidney disease. On the other hand, the CG + GG genotypes are independently related to LVH in end-stage kidney disease patients.
